# Metabolic engineering strategies for optimizing acetate reduction, ethanol yield and osmotolerance in S*accharomyces cerevisiae*

**DOI:** 10.1186/s13068-017-0791-3

**Published:** 2017-04-26

**Authors:** Ioannis Papapetridis, Marlous van Dijk, Antonius J. A. van Maris, Jack T. Pronk

**Affiliations:** 1 Industrial Microbiology Section, Department of Biotechnology, Delft University of Technology, Van der Maasweg 9, 2629 HZ Delft, The Netherlands; 2 Division of Industrial Biotechnology, Department of Biology and Biological Engineering, Chalmers University of Technology, Kemivägen 10, 41296 Gothenburg, Sweden; 3 Division of Industrial Biotechnology, School of Biotechnology, KTH Royal Institute of Technology, AlbaNova University Centre, 10691 Stockholm, Sweden

**Keywords:** Yeast, NADH, NADPH, Redox engineering, Acetic acid, Osmotic stress, Bioethanol

## Abstract

**Background:**

Glycerol, whose formation contributes to cellular redox balancing and osmoregulation in *Saccharomyces cerevisiae*, is an important by-product of yeast-based bioethanol production. Replacing the glycerol pathway by an engineered pathway for NAD^+^-dependent acetate reduction has been shown to improve ethanol yields and contribute to detoxification of acetate-containing media. However, the osmosensitivity of glycerol non-producing strains limits their applicability in high-osmolarity industrial processes. This study explores engineering strategies for minimizing glycerol production by acetate-reducing strains, while retaining osmotolerance.

**Results:**

*GPD2* encodes one of two *S. cerevisiae* isoenzymes of NAD^+^-dependent glycerol-3-phosphate dehydrogenase (G3PDH). Its deletion in an acetate-reducing strain yielded a fourfold lower glycerol production in anaerobic, low-osmolarity cultures but hardly affected glycerol production at high osmolarity. Replacement of both native G3PDHs by an archaeal NADP^+^-preferring enzyme, combined with deletion of *ALD6*, yielded an acetate-reducing strain the phenotype of which resembled that of a glycerol-negative *gpd1Δ gpd2Δ* strain in low-osmolarity cultures. This strain grew anaerobically at high osmolarity (1 mol L^−1^ glucose), while consuming acetate and producing virtually no extracellular glycerol. Its ethanol yield in high-osmolarity cultures was 13% higher than that of an acetate-reducing strain expressing the native glycerol pathway.

**Conclusions:**

Deletion of *GPD2* provides an attractive strategy for improving product yields of acetate-reducing *S. cerevisiae* strains in low, but not in high-osmolarity media. Replacement of the native yeast G3PDHs by a heterologous NADP^+^-preferring enzyme, combined with deletion of *ALD6*, virtually eliminated glycerol production in high-osmolarity cultures while enabling efficient reduction of acetate to ethanol. After further optimization of growth kinetics, this strategy for uncoupling the roles of glycerol formation in redox homeostasis and osmotolerance can be applicable for improving performance of industrial strains in high-gravity acetate-containing processes.

**Electronic supplementary material:**

The online version of this article (doi:10.1186/s13068-017-0791-3) contains supplementary material, which is available to authorized users.

## Background

By functionally replacing fossil-fuel-derived compounds, microbial production of chemicals and transport fuels can contribute to a transition to a sustainable, low-carbon global economy [1]. The total industrial production of fuel ethanol, which reached ca. 100 billion litres in 2015, is predicted to increase further [2]. The yeast *Saccharomyces cerevisiae* is the established microbial cell factory for conversion of starch- and sucrose-derived hexose sugars to ethanol, as it combines a high ethanol yield and productivity with robustness under process conditions [3–5]. Efforts in yeast strain improvement and process optimization of corn–starch and cane–sugar-based bioethanol productions have further improved product yields and productivity [5]. Furthermore, intensive metabolic and evolutionary engineering studies have yielded yeast strains capable of efficiently fermenting the pentose sugars xylose and arabinose, thus paving the way for yeast-based ‘second-generation’ bioethanol production from lignocellulosic hydrolysates [6–8].

In industrial bioethanol production, the carbohydrate feedstock represents the single largest cost factor [9]. Maximizing ethanol yield on sugar is therefore a key requirement, especially in second-generation processes, ethanol yields and productivity of which are generally still lower than those of first-generation processes [6–8]. Adequate yeast performance in lignocellulosic hydrolysates also requires tolerance to inhibitors that are released during biomass pre-treatment and hydrolysis [10–12]. Suboptimal ethanol yields in industrial processes are caused by formation of biomass and low-molecular-weight metabolites, of which glycerol accounts for a loss of up to 4% of the carbohydrate substrate [13]. Under anaerobic conditions, wild-type *S. cerevisiae* strains require glycerol formation to re-oxidize NADH formed during biosynthesis or during production of metabolites whose formation results in net NADH formation [14, 15]. As the major compatible solute in *S. cerevisiae*, glycerol also plays a key role in osmotolerance [16, 17].

In *S. cerevisiae,* glycerol formation is initiated by reduction of the glycolytic intermediate dihydroxyacetone phosphate to glycerol-3-phosphate, a reaction catalysed by two isoenzymes of NAD^+^-dependent glycerol-3P dehydrogenase (G3PDH), Gpd1 and Gpd2 [18]. Glycerol-3P is subsequently hydrolysed to glycerol and inorganic phosphate by glycerol-3P phosphatase, isoenzymes of which are encoded by *GPP1* and *GPP2*. Reoxidation of 1 mol of NADH through glycerol production requires 0.5 mol glucose and 1 mol ATP [18]. Several metabolic engineering strategies have demonstrated that altering redox-cofactor specificity of reactions in biosynthesis [13] or in sugar dissimilation [19] can be used to decrease glycerol formation from sugars.

*GPD1* and *GPD2* are differentially regulated, as transcriptional upregulation of *GPD1* mainly occurs in response to osmotic stress, while regulation of *GPD2* is linked to redox homeostasis [20–22]. Complete elimination of glycerol production by inactivation of both *GPD1* and *GPD2* has been reported to abolish anaerobic growth and to greatly increase osmosensitivity [20, 21, 23]. Anaerobic growth of *gpd1Δ gpd2Δ* cultures can be restored by addition of an external electron acceptor, such as acetoin, to growth media [23]. Recently, *S. cerevisiae* strains have been engineered to use acetic acid or CO_2_ as external electron acceptors [24–28]. Functional expression of a heterologous acetylating-acetaldehyde dehydrogenase (A-ALD), together with the activities of the native acetyl-CoA synthetases and alcohol dehydrogenases, enabled the NADH-dependent reduction of acetic acid to ethanol [25]. CO_2_ is abundantly present in all industrial ethanol fermentation processes, while acetic acid is an important, ubiquitous inhibitor of yeast performance in lignocellulosic hydrolysates [10, 11]. Use of acetic acid as an external ‘redox sink’ is highly attractive for second-generation bioethanol processes, since its reduction to ethanol not only increases product yields, but simultaneously contributes to detoxification of the fermentation medium [25, 29, 30].

Although metabolic engineering has enabled the replacement of glycerol production by the reduction of acetic acid to ethanol, the increased osmosensitivity of *gpd1Δ gpd2Δ* strains has not been fully resolved. Process intensification of bioethanol production via the introduction of high-gravity fermentation processes will make osmotolerance ever more important [31–36]. Previous research on improving osmotolerance of *gpd1Δ gpd2Δ* strains explored production of alternative compatible solutes, including the polyols mannitol and sorbitol [37], trehalose [38–40] and proline [41, 42]. These alternative compounds, however, did not confer the same osmotolerance as glycerol. Evolutionary engineering of an acetate-reducing *gpd1Δ gpd2Δ* strain yielded a strain that could grow anaerobically at 1 mol L^−1^ glucose without loss of acetate-reduction potential or ethanol yield [43]. However, the underlying genetic changes were not fully resolved [43]. Tuning the expression of the native G3PDH genes can lead to decreased glycerol production, but the resulting strains still require the production of considerable amounts of this by-product to maintain the redox-cofactor balance [34, 35, 44]. To uncouple the roles of glycerol formation in NADH reoxidation and osmotolerance in *S. cerevisiae*, it would be of interest to alter the redox-cofactor specificity of G3PDH. Specifically, making this reaction NADPH-dependent might uncouple glycerol production from NAD^+^ regeneration. In such a scenario, NAD^+^ could then be exclusively regenerated via acetate or CO_2_ reduction. Simultaneously, NADPH-dependent formation of intracellular glycerol would enable the synthesis of a compatible solute with minimal losses in ethanol yield. Recently, an NADP^+^-preferring G3PDH, encoded by the *gpsA* gene from the thermophilic archaeon *Archaeoglobus fulgidus* was described [45]. Based on its unusual cofactor preference and, due to its thermophilic origin, anticipated low in vivo activity in yeast, we hypothesized that *A. fulgidus* GpsA might be an interesting candidate to replace the NAD^+^-dependent Gpd1 and Gpd2 enzymes in *S. cerevisiae*.

The goal of this study is to explore new metabolic engineering strategies for construction of acetate-reducing, osmotolerant *S. cerevisiae* strains with a minimal, non-zero production of glycerol. To this end, acetate-reducing strains with different configurations of the native glycerol production pathway, as well as strains in which *GPD1* and *GPD2* were replaced by *A. fulgidus gpsA*, were constructed. To construct acetate-reducing strains, the ethanolamine utilization protein of *E. coli*, encoded by *eutE* [Genbank: WP_001075673.1], was overexpressed, as it was previously shown to support near-wild-type anaerobic growth rates in a *gpd1Δ gpd2Δ* strain background [29]. The impacts of these engineering strategies on acetate reduction and glycerol production were quantitatively analysed in anaerobic bioreactor batch cultures grown on low- and high-osmolarity media.

## Methods

### Strain propagation and maintenance

All *S. cerevisiae* strains used in this study belong to the CEN.PK lineage [46] (Table 1). *S. cerevisiae* cultures were propagated in synthetic medium [47] containing 20 g L^−1^ glucose. *E. coli DH5a* cultures for plasmid cloning were propagated in LB medium (10 g L^−1^ Bacto tryptone, 5 g L^−1^ Bacto yeast extract, 5 g L^−1^ NaCl) containing 100 mg L^−1^ ampicillin. All strains were stored at −80 °C, after addition of sterile glycerol (30% v v^−1^) to growing cultures.

**Table 1:** *Saccharomyces cerevisiae* strains used in this study

Strain name	Relevant genotype	Origin
IMX585	*MAL2*-*8c SUC2 can1::cas9*-*natNT2*	[48]
IMX581	*ura3*-*52 MAL2*-*8c SUC2 can1::cas9*-*natNT2*	[48]
IMZ160	*ura3 leu2::LEU2* [pRS405] *gpd1::loxP gpd2::hphMX4* pUDE43	[43]
IME324	*ura3*-*52 MAL2*-*8c SUC2 can1::cas9*-*natNT2* p426-*TEF* (empty)	This work
IMX884	*ura3*-*52 MAL2*-*8c SUC2 can1::cas9*-*natNT2 gpd2::eutE* pROS10 (*GPD2*-targeting)	This work
IMX992	*ura3*-*52 MAL2*-*8c SUC2 can1::cas9*-*natNT2 sga1::eutE* pUDR119	This work
IMX776	*ura3*-*52 MAL2*-*8c SUC2 can1::cas9*-*natNT2 gpd1::gpsA gpd2::eutE* pUDR240	This work
IMX901	*ura3*-*52 MAL2*-*8c SUC2 can1::cas9*-*natNT2 gpd1::gpsA gpd2::eutE ald6Δ* pUDR240	This work
IMX888	*MAL2*-*8c SUC2 can1::cas9*-*natNT2 gpd1Δ gpd2::eutE*	[29]
IMX900	*MAL2*-*8c SUC2 can1::cas9*-*natNT2 gpd1Δ gpd2::eutE ald6Δ*	This work
IMX1039	*ura3*-*52 MAL2*-*8c SUC2 can1::cas9*-*natNT2 gpd1::gpsA gpd2::eutE ald6Δ*	This work
IMX1120	*MAL2*-*8c SUC2 can1::cas9*-*natNT2 gpd1Δ gpd2::eutE ald6Δ sga1::ALD6*	This work
IMX1142	*ura3*-*52 MAL2*-*8c SUC2 can1::cas9*-*natNT2 gpd1::gpsA gpd2::eutE ald6Δ sga1::ALD6* pUDR103	This work

### Construction of expression cassettes and plasmids

Plasmids used in this study are listed in Table 2. Plasmids-expressing chimeric gRNAs were used for CRISPR/Cas9-mediated genome editing [48]. Unique Cas9-recognition sequences in *GPD1*, *GPD2*, *SGA1* and *ALD6* were selected as described previously [29]. PCR for construction of expression cassettes and diagnostic PCR were performed using Phusion Hot Start II High Fidelity DNA Polymerase and Dreamtaq polymerase (Thermo Scientific, Waltham, MA), respectively, according to the manufacturer’s guidelines. For the construction of pUDR240, the backbone of the plasmid was PCR amplified using the double-binding primer 5793 (Additional file 1) and pROS10 as template. The insert fragment, expressing the *GPD1*-targeting and *GPD2*-targeting gRNA cassettes, was amplified using primers 6965–6966 and pROS10 as template. For the construction of pUDR103, the plasmid backbone of pMEL10 was PCR amplified using primers 5792–5980. The *SGA1*-targeting gRNA expression cassette was PCR amplified using primers 5979–7023 and pMEL10 as template. For the construction of pUDR264, the plasmid backbone of pMEL11 was PCR amplified using primers 5792–5980. The *ALD6*-targeting gRNA expression cassette was PCR amplified using primers 5979–7610 and pMEL11 as a template. Plasmids were assembled with the Gibson Assembly Cloning kit (New England Biolabs, Ipswich, MA), after downscaling the supplier’s protocol to 10 μL reaction volumes. Plasmids pUDR240 and pUDR264 were cloned in *E. coli DH5a* cells after transformation by electroporation and plasmid re-isolation with a miniprep kit (Sigma-Aldrich, St. Louis, MO). Correct clones were verified by restriction digestion or by diagnostic PCR (DreamTaq polymerase, Additional file 1). For the single deletion of *GPD2*, a plasmid backbone was PCR amplified with the double-binding primer 5793 and pROS10 as template. The insert fragment, expressing two identical *GPD2*-targeting gRNA cassettes, was amplified using primer 6966 and pROS10 as templates. For the single deletion of *GPD2*, the two plasmid fragments were transformed directly into yeast cells and assembled in vivo.

**Table 2:** Plasmids used in this study

Plasmid	Characteristics	Origin
p426-*TEF* (empty)	2 μ, *URA3*, *TEF1p*-*CYC1t*	[75]
pMEL10	2 μ, *KlURA3*, *SNR52*p-gRNA.*CAN1*.Y-*SUP4*t	[48]
pMEL11	2 μ, *amdS*, *SNR52*p-gRNA.*CAN1*.Y-*SUP4*t	[48]
pROS10	*KlURA3*-gRNA.*CAN1*-2mu-gRNA.*ADE2*	[48]
pUDI076	pRS406-*TDH3*p-*eutE*-*CYC1t*	[29]
pUDR103	2 μ, *KlURA3*, *SNR52*p-gRNA.*SGA1*.Y-*SUP4*t	This work
pUDR119	2 μ, *amdS*, *SNR52*p-gRNA.*SGA1*.Y-*SUP4*t	[76]
pUDR240	*KlURA3*-gRNA.*GPD1*-2mu-gRNA.*GPD2*	This work
pUDR264	2 μ, *amdS*, *SNR52p*-*gRNA.ALD6.Y*-*SUP4t*	This work
pMK-RQ-*gpsA*	Delivery vector, codon-optimized *gpsA* ORF	GeneArt, Germany

An *S. cerevisiae* codon-optimized version of *A. fulgidus gpsA* [Genebank: AAB90367.1], based on the codon preference of highly expressed yeast glycolytic genes [49], was synthesized by GeneArt GmbH (Regensburg, Germany) [Genebank: KY554758]. An integration cassette for replacing the coding region of *GPD1* by the codon-optimized *gpsA* sequence was PCR amplified with primers 7862–7863 and pMK-RQ-*gpsA* as template. Codon-optimized expression cassettes for the *E. coli* EutE acetylating-acetaldehyde dehydrogenase gene (*TDH3*p-*eutE*-*CYC1t*), aimed at integration in the *GPD2* or *SGA1* locus, were amplified with primers 7991–7992 or 7211–7025, respectively, using pUDI076 [29] as a template. A cassette expressing *ALD6* from its native promoter and terminator sequences, aimed at integration in the *SGA1* locus, was amplified with primers 9809–9810, using genomic DNA of *S. cerevisiae* IMX581 as a template. Integration cassettes were flanked by 60-bp sequences that enabled integration by homologous recombination after CRISPR/Cas9-mediated introduction of double-strand breaks in selected *S. cerevisiae* genomic loci.

### Strain construction

The lithium acetate/polyethylene glycol method [50] was used for yeast transformation. After transformation with plasmids pUDR103, pUDR240 and after single deletion of *GPD2*, transformants were selected on synthetic medium agar plates [47] containing 20 g L^−1^ glucose. After transformation with plasmids pUDR119 and pUDR264, selection and counter selection were performed as described [51]. Counter selection of plasmids carrying *URA3* was performed on YP agar plates (10 g L^−1^ Bacto yeast extract, 20 g L^−1^ Bacto peptone) supplemented with glucose (20 g L^−1^ final concentration) and 5-fluoroorotic acid (1 g L^−1^ final concentration). Diagnostic colony PCR was used for genotypic analysis of selected colonies. Co-transformation of pUDR119 and the *SGA1*-flanked *TDH3p*-*eutE*-*CYC1t* cassette into strain IMX581 yielded strain IMX992, in which *eutE* was overexpressed in the presence of functional *GPD1* and *GPD2* genes. Co-transformation of the two fragments of the *GPD2*-targeting gRNA plasmid and the *GPD2*-flanked *TDH3p*-*eutE*-*CYC1t* cassette to strain IMX581 yielded strain IMX884, in which *GPD2* was deleted and *eutE* was overexpressed. Co-transformation of pUDR240, the *GPD1*-flanked *gpsA* coding sequence and the *GPD2*-flanked *TDH3p*-*eutE*-*CYC1t* cassette to strain IMX581 yielded strain IMX776, in which *gpsA* was expressed from the native *GPD1* promoter and terminator, *GPD2* was deleted and *eutE* was overexpressed. Co-transformation of pUDR264 and the repair oligonucleotides 7608–7609, followed by pUDR264 counter selection, into strains IMX776 and IMX888 yielded strains IMX901 and IMX900, respectively, in which *ALD6* was deleted. Counter selection of pUDR240 from IMX901 yielded strain IMX1039. Strain IMX1142, in which the native *ALD6* gene was re-introduced in the *SGA1* locus, was obtained by co-transformation of pUDR103 and the *SGA1*-flanked *ALD6* cassette into strain IMX1039. Co-transformation of pUDR119 and the *SGA1*-flanked *ALD6* cassette into strain IMX900, following pUDR119 counter selection, yielded strain IMX1120. The empty-vector reference strain IME324 was obtained by transformation of IMX581 with p426-*TEF*.

### Bioreactor batch cultivation

Anaerobic batch cultures were grown in 2-L bioreactors (Applikon, Schiedam, The Netherlands) on synthetic medium [47] supplemented with acetic acid (3 g L^−1^ final concentration). In high-osmolarity cultures of the acetate-consuming strains IMX776, IMX901 and IMX1142, the concentration of acetic acid was re-set to 3 g L^−1^ when it reached a value below 1.5 g L^−1^, with the addition of glacial acetic acid, to prevent acetic-acid limitation [43]. After autoclaving the mineral salt components of the synthetic medium and acetic acid at 120 °C for 20 min, anaerobic growth media were supplemented with sterile antifoam C (0.2 g L^−1^) (Sigma-Aldrich), ergosterol (10 mg L^−1^), Tween 80 (420 mg L^−1^) and filter-sterilized vitamin solution [47]. Glucose solutions were autoclaved separately at 110 °C for 20 min and added to low- and high-osmolarity media at final concentrations of 20 and 180 g L^−1^ (1 M), respectively. Shake-flask cultures (100 mL) were inoculated with frozen glycerol stock cultures (1 mL) and grown on synthetic medium supplemented with glucose (20 g L^−1^ final concentration). Samples from these cultures were used as inocula for 100 mL shake-flask pre-cultures on the same medium, yielding an initial OD_660_ of 0.1–0.3. Upon reaching mid-exponential phase (OD_660_ 4–6), samples from these pre-cultures were used to inoculate anaerobic bioreactor cultures, yielding an initial OD_660_ of 0.15–0.2. Anaerobic conditions were maintained by continuously sparging nitrogen gas (<10 ppm oxygen) at a rate of 0.5 L min^−1^. Norprene tubing and Viton O-rings were used to minimize oxygen diffusion into the reactors. In low-osmolarity cultures, the culture pH was automatically controlled at 5.0 by addition of 2 M KOH. In high-osmolarity cultures (pH 5.0), 12.5% v v^−1^ NH_4_OH solution was used as titrant to prevent nitrogen limitation. The stirrer speed was set at 800 rpm, and temperature was controlled at 30 °C. Evaporation was minimized by cooling the outlet gas to 4 °C in a condenser.

### Enzyme-activity assays

Cell extracts were prepared by sonication [52] of biomass from exponential-phase shake-flask cultures (OD_660_ 5–6) grown on synthetic medium (20 g L^−1^ glucose) in an aerobic incubator. Enzyme-activity assays were performed at 30 °C by continuous spectrophotometric monitoring of the conversion of NAD(P)H to NAD(P)^+^ at 340 nm. For the determination of acetylating-acetaldehyde dehydrogenase activity [25], cells were sonicated in 100 mM potassium-phosphate buffer (KPB, pH 7.5) with 2 mM MgCl_2_ and 1 mM dithiothreitol. The 1-mL reaction mixture contained 50 mM KPB (pH 7.5), 0.15 mM NADH and 50 or 70 μL cell extract. Reactions were started by addition of acetyl-CoA to a final concentration of 0.5 mM. To assess if expression of *A. fulgidus gpsA* resulted in a change in the cofactor preference of glycerol-3-phosphate dehydrogenase (G3PDH) in *S. cerevisiae*, G3PDH activities were measured by means of a modified version of a published assay optimized for GpsA [45]. In the modified assay, 20 mM Tris–HCl (pH 8.2) buffer supplemented with 10 mM EDTA was used for harvesting and storage of cells, and sonication was done in 20 mM Tris–HCl (pH 8.2) buffer with 2 mM EDTA. The 1-mL reaction mixture contained 50 mM Tris–HCl (pH 6.6), 2 mM EDTA, 0.15 mM NADH or NADPH and 50 or 70 μL cell extract. The reaction was started by addition of dihydroxyacetone phosphate to a final concentration of 4 mM. All assays were performed on samples from two independent cultures, and enzyme activities were proportional to the volume of cell extract added to the assay.

### Intracellular glycerol determination

Shake-flask pre-cultures on synthetic medium (20 g L^−1^ glucose) were inoculated from frozen stocks. After reaching mid-exponential phase, cells were washed with sterile demineralized water and used as inoculum for anaerobic shake-flask cultures on the same medium as the high-osmolarity bioreactor batch cultivations. Anaerobic shake-flask cultures were grown in a Bactron anaerobic chamber (Sheldon Manufacturing, Cornelius, OR) at 30 °C. Mid-exponential-phase cultures were harvested and centrifuged at 4000×*g* for 5 min. The supernatant was discarded, and the cells were resuspended in 0.005 mol L^−1^ H_2_SO_4_ and incubated at 100 °C for 5 min. The cell suspension was centrifuged at 4000×*g* for 5 min, and the supernatant was used for HPLC analysis. For the calculation of the pellet volume, an average density of the pellet of 1.1 g mL^−1^ was used [53]. For the conversion of intracellular glycerol concentration from g (g dry weight)^−1^ to g L^−1^, an intracellular volume of 2.6 mL (g dry weight)^−1^ was used [54].

### Analytical methods

Biomass dry weight determination, HPLC analysis of extracellular metabolites and correction for ethanol evaporation were performed as previously described [25]. Culture offgas composition was analysed as previously described [25], except for batch cultures grown under high-osmolarity conditions with strains IMX992, IMX884, IMX776 and IMX901, in which production of CO_2_ was calculated from ethanol production, assuming formation of 1 mol CO_2_ per mol ethanol produced. Prior to glucose and ethanol concentration measurements in high-osmolarity fermentations, culture supernatant was diluted 1:1 with demineralized water. Product yields and ratios in batch cultures were calculated from a minimum of five samples taken during the mid-exponential growth phase [29]. Biomass concentrations corresponding to samples taken before the mid-exponential growth phase (OD_660_ < 1) were calculated based on OD_660_ measurements, using calibration curves based on a minimum of five samples taken in mid-exponential phase for which biomass dry weight and OD_660_ were measured [29].

## Results

### Limited impact of the expression of an acetate-reduction pathway in *GPD1 GPD2 S. cerevisiae*

**Fig. 1:**
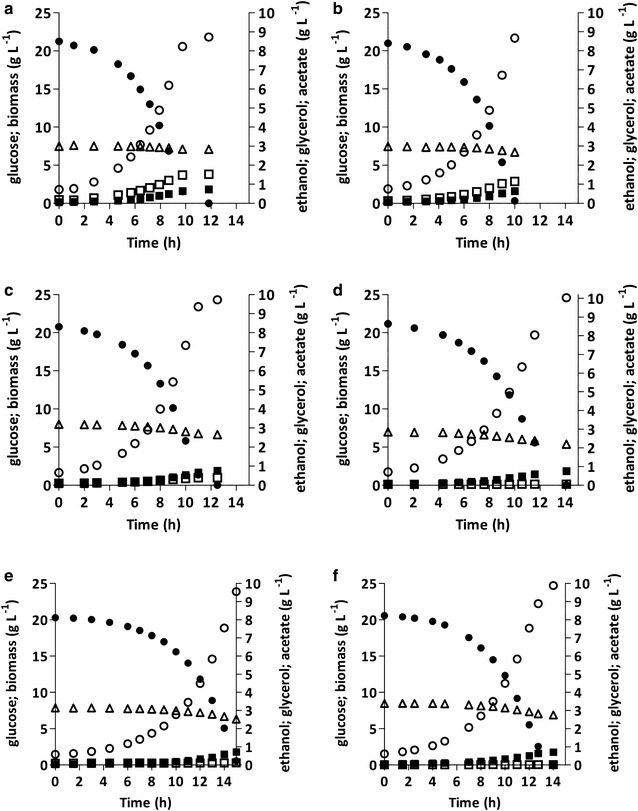
Growth, glucose consumption and product formation in anaerobic bioreactor batch cultures of *S. cerevisiae* strains with different genetic modifications in glycerol and acetate metabolism. Cultures were grown on synthetic medium containing 20 g L^−1^ glucose and 3 g L^−1^ acetic acid (pH 5). **a** strain IME324 (*GPD1 GPD2*); **b** strain IMX992 (*GPD1 GPD2 sga1::eutE*); **c** strain IMX884 (*GPD1 gpd2::eutE*); **d** strain IMX776 (*gpd1::gpsA gpd2::eutE*); **e** strain IMX901 (*gpd1::gpsA gpd2::eutE ald6Δ*); **f** strain IMX888 (*gpd1Δ gpd2::eutE*). *Closed circle* glucose; *closed square* biomass; *open square* glycerol; *open circle* ethanol; *open triangle* acetate. **a**–**f** display single representative cultures from a set of two independent duplicate cultures for each strain. Data on strain IMX888 were taken from [29]

Previous studies on acetate reduction by anaerobic, glucose-grown *S. cerevisiae* cultures were based on *gpd1Δ gpd2Δ* strains [25, 29]. In these strains, the role of the native glycerol pathway in NADH reoxidation was entirely replaced by reduction of externally supplied acetate to ethanol. To investigate the impact of co-expressing an acetate-reduction pathway with a fully functional glycerol pathway, growth and product formation of strain IMX992 (*GPD1 GPD2 sga1::eutE*) were analysed in anaerobic, glucose-grown bioreactor batch cultures on 20 g L^−1^ glucose, supplemented with 3 g L^−1^ acetic acid (Fig. 1; Table 3) and compared with the acetate non-reducing reference strain IME324. Under these conditions and consistent with previous reports [29, 43], IME324 (*GPD1 GPD2*) showed an acetate consumption of 2.43 mmol (g biomass)^−1^ (Table 3). In acetate non-reducing strains, consumption of small amounts of acetate can reflect intracellular accumulation and/or use of extracellular-acetate-derived acetyl-CoA as a biosynthetic precursor [55]. Strain IMX992 (*GPD1 GPD2 sga1::eutE*) showed an acetate consumption of 3.35 mmol (g biomass)^−1^, which was only 0.92 mmol (g biomass)^−1^ higher than the acetate consumption by the *GPD1 GPD2* reference strain. Conversely, under identical conditions, strain IMX888 (*gpd1Δ gpd2::eutE*) showed an acetate consumption of 6.92 mmol (g biomass)^−1^ in a previous study [29]. Consistent with its marginally higher acetate consumption, glycerol production by strain IMX992 decreased only slightly, from 9.19 to 8.28 mmol glycerol (g biomass)^−1^, relative to strain IME324 (Table 3). Clearly, in glucose-fermenting engineered *S. cerevisiae* strains, EutE-based acetate reduction could not efficiently compete for NADH with a fully functional native glycerol pathway.

**Table 3:** Specific growth rates (*μ*) and stoichiometric relationships between glycerol production and biomass formation, acetate consumption and glucose consumption, and acetate consumption and biomass formation in anaerobic bioreactor batch cultures of *S. cerevisiae* strains with different genetic modifications in glycerol and acetate metabolism

Strain	IME324	IMX992	IMX884	IMX776	IMX901	IMX888^a^
Relevant genotype	*GPD1* *GPD2*	*GPD1* *GPD2* *sga1::eutE*	*GPD1* *gpd2::eutE*	*gpd1::gpsA* *gpd2::eutE*	*gpd1::gpsA* *gpd2::eutE* *ald6Δ*	*gpd1Δ* *gpd2::eutE*
*μ* (h^−1^)	0.31 ± 0.01	0.30 ± 0.00	0.31 ± 0.01	0.24 ± 0.01	0.24 ± 0.01	0.26 ± 0.01
Ratio glycerol produced/biomass [mmol (g biomass)^−1^]	9.19 ± 0.08	8.28 ± 0.14	1.92 ± 0.06	<0.1	<0.1	<0.1
Ratio acetate consumed/biomass [mmol (g biomass)^−1^]	2.43 ± 0.16	3.35 ± 0.08	5.77 ± 0.25	6.66 ± 0.01	6.41 ± 0.28	6.92 ± 0.12
Ratio acetate consumed/glucose (g g^−1^)	0.010 ± 0.000	0.015 ± 0.000	0.026 ± 0.001	0.031 ± 0.001	0.031 ± 0.000	0.032 ± 0.000

Cultures were grown on synthetic medium containing 20 g L^−1^ glucose and 3 g L^−1^ acetic acid (pH 5). Specific growth rates and stoichiometries were calculated from the mid-exponential growth phase and represent averages ± mean deviations of data obtained from independent duplicate cultures. In all cultures, carbon recoveries were between 95 and 100%. Enzyme activities of acetylating-acetaldehyde dehydrogenase in cell extracts of *eutE*-expressing strains were similar (Additional file 2)

^a^Data on strain IMX888 were taken from [29]

### Deletion of *GPD2* improves acetate reduction by an *eutE*-expressing strain

*GPD2* encodes the redox-regulated isoenzyme of G3PDH and its deletion has been reported to cause impaired anaerobic growth of *S. cerevisiae* [23, 35]. In acetate-supplemented anaerobic cultures of strain IMX884 (*GPD1 gpd2::eutE*), *eutE* expression fully compensated for the absence of a functional Gpd2 enzyme, both in terms of specific growth rate and in terms of biomass yield on glucose (Table 3; Fig. 1; Additional file 3). Compared to strain IMX992 (*GPD1 GPD2 sga1::eutE*), strain IMX884 showed a fourfold lower production of glycerol [1.92 and 8.28 mmol glycerol (g biomass)^−1^, respectively] and a correspondingly higher EutE-based acetate consumption [3.34 and 0.92 mmol acetate (g biomass)^−1^, respectively], corrected for acetate consumption by the acetate non-reducing reference strain IME324, resulting in an ethanol yield on glucose of 0.46 g g^−1^ (Additional file 3). These results indicate that, at least in low-osmolarity media, inactivation of *GPD2* enables the EutE-based acetate-reduction pathway to efficiently compete for redox equivalents with the glycerol pathway. This engineering strategy not only resulted in a markedly higher acetate consumption, but also in a higher ethanol yield on glucose than observed in the acetate non- reducing reference strain IME324 (Table 3; Additional file 3).

### Functional expression of an NADPH-preferring G3PDH in *S. cerevisiae*

As outlined above, expression of the NADP^+^-preferring G3PDH encoded by *A. fulgidus gpsA* might enable strategies to uncouple the roles of glycerol metabolism in yeast osmotolerance and redox balancing. To investigate whether *gpsA* can be functionally expressed in *S. cerevisiae*, its coding sequence was codon optimized for expression in yeast and integrated at the *GPD1* locus of strain IMX581 (along with integration of *eutE* at the *GPD2* locus), yielding strain IMX776 (*gpd1::gpsA gpd2::eutE*). This insertion was designed to place *gpsA* under the control of the *GPD1* promoter and terminator, in order to enable upregulation of its expression at high-osmolarity [20, 21].

**Fig. 2:**
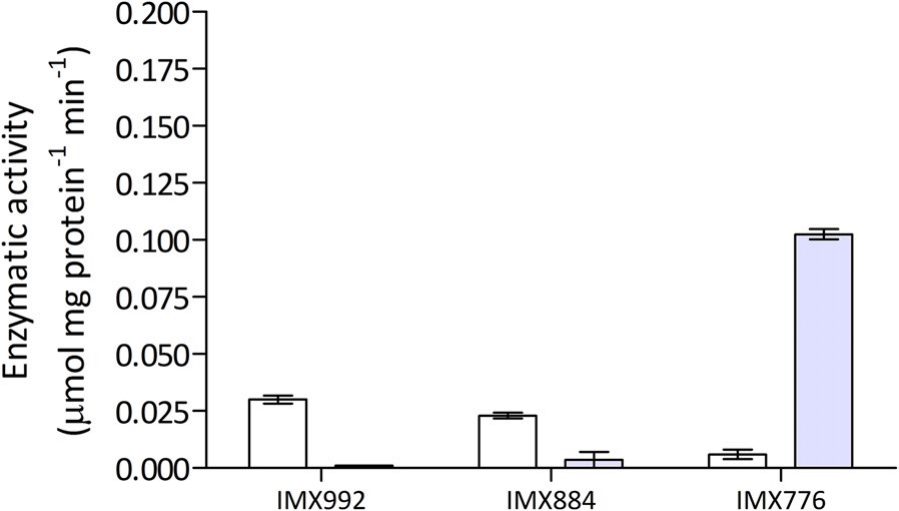
Specific rates of NADH-dependent (*white bars*) and NADPH-dependent (*blue bars*) reduction of dihydroxyacetone phosphate by cell extracts of shake-flask cultures on synthetic medium (20 g L^−1^ glucose) of *S. cerevisiae* strains IMX992 (*GPD1 GPD2*), IMX884 (*GPD1 gpd2::eutE*) and IMX776 (*gpd1::gpsA gpd2::eutE*). Data represent averages ± mean deviations of assays on independent duplicate cultures

Enzyme-activity assays in cell extracts showed that, in strain IMX776, replacement of the native *GPD1* and *GPD2* genes by *gpsA* resulted in a switch in cofactor preference of glycerol-3-phosphate dehydrogenase (G3PDH, Fig. 2). The *gpsA*-expressing strain showed in vitro activities of 0.103 ± 0.004 and 0.006 μmol mg protein^−1^ min^−1^ with NADPH and NADH, respectively. As a result, the ratio of NADPH- and NADH-linked rates of dihydroxyacetone phosphate reduction was ca. 500-fold higher in strain IMX776 than in the reference strain IMX992, which expresses the native *GPD1* and *GPD2* genes. These observations are consistent with a previous report on the cofactor preference of GpsA, expressed in *E. coli* [45], and show that the enzyme was functionally expressed in yeast.

### Increased acetate reduction and decreased glycerol production in a *gpsA*-expressing yeast strain

The combined activity of acetyl-CoA synthetase and EutE, both of which are essential for acetate reduction and Ald6, the major NADP^+^-dependent, cytosolic isoform of acetaldehyde dehydrogenase [56], could potentially form an ATP-driven dehydrogenase cycle (NADH + NADP^+^ → NADPH + NAD^+^ [29]). In *gpsA*-expressing strains, NADPH formed via this cycle might increase glycerol production and, consequently, decrease ethanol yields (Additional file 8). Therefore, *ALD6* was deleted in the *gpsA*-expressing, acetate-reducing strain IMX776 (*gpd1::gpsA gpd2::eutE*), yielding strain IMX901.

In anaerobic, acetate-supplemented bioreactor batch cultures, the specific growth rate of strain IMX776 (*gpd1::gpsA gpd2::eutE*) was 0.24 h^−1^, which was ca. 20% lower than that of the reference strain IME324 (*GPD1 GPD2*). The physiology of strain IMX776 in these anaerobic low-osmolarity cultures, including the stoichiometry of biomass formation and acetate consumption, closely resembled that of strain IMX888 (*gpd1Δ gpd2::eutE*) (Table 3; Fig. 1; Additional file 3). Virtually no extracellular glycerol was formed by strain IMX776, indicating that, under these conditions, the in vivo activity of NADPH-dependent glycerol production in this strain was minimal. Consistent with this notion, growth and product formation in anaerobic cultures of strain IMX901 (*gpd1::gpsA gpd2::eutE ald6Δ)* was similar to the observed performance of strains IMX776 or IMX888 under these conditions.

### Growth at high-osmolarity negatively affects acetate reduction by a *gpd2Δ* strain

**Fig. 3:**
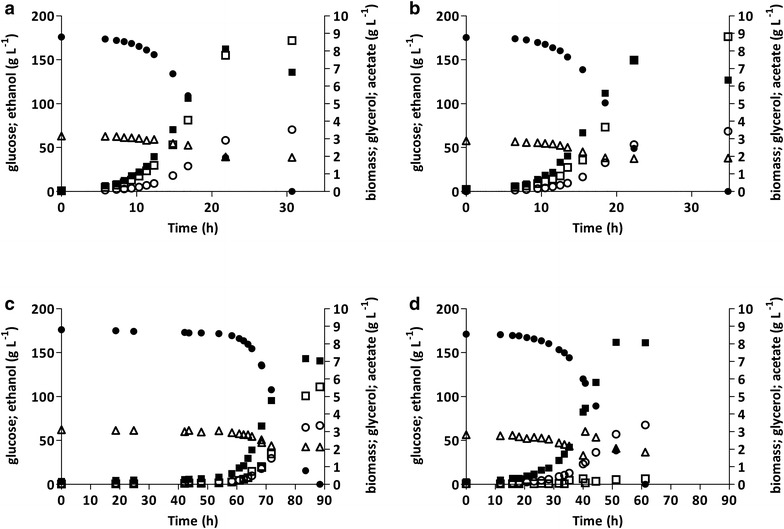
Growth, glucose consumption and product formation in anaerobic bioreactor batch cultures of *S. cerevisiae* strains with different genetic modifications in glycerol and acetate metabolism. Cultures were grown on synthetic medium containing 180 g L^−1^ glucose and 3 g L^−1^ acetic acid (pH 5). **a** Strain IMX992 (*GPD1 GPD2 sga1::eutE*); **b** strain IMX884 (*GPD1 gpd2::eutE*); **c** strain IMX776 (*gpd1::gpsA gpd2::eutE*); **d** strain IMX901 (*gpd1::gpsA gpd2::eutE ald6Δ*). *Closed circle* glucose; *closed square* biomass; *open square* glycerol; *open circle* ethanol; *open triangle* acetate. **a**–**c** display single representative cultures from a set of two independent duplicate cultures for each strain. In the culture of IMX901, acetic acid was added externally immediately after the exponential growth phase was finished

To assess the impact of high-osmolarity on the acetate reduction observed in the *GPD1 gpd2::eutE* strain IMX884, its performance was compared with that of strain IMX992 (*GPD1 GPD2 sga1::eutE*) in anaerobic bioreactor batch cultures grown on 1 mol L^−1^ (180 g L^−1^) glucose. In contrast to the low-osmolarity cultures, in which strains continued to grow exponentially until glucose was depleted (Fig. 1), high-osmolarity conditions showed a biphasic growth profile, in which the exponential phase was followed by second, slower growth phase (Fig. 3). This biphasic growth profile probably reflects a nutritional limitation other than carbon source depletion. For example, concentrations of the anaerobic growth factors Tween-80 and ergosterol were not increased in high-osmolarity media to avoid potential toxic effects [15]. A similar growth pattern in high-glucose cultures has been reported previously [43].

Consistent with previously reported data on a congenic, non-acetate-reducing *GPD1 GPD2* strain grown under high-osmolarity conditions [43], the initial specific growth rate of strain IMX992 (*GPD1 GPD2 sga1::eutE*) was not affected by increasing the glucose concentration in the medium to 1 mol L^−1^ (Tables 3, 4). Acetate consumption in the high-osmolarity cultures by this strain was even slightly lower than observed during growth on 20 g L^−1^ glucose [2.67 and 3.35 mmol (g biomass^−1^), respectively]. This observation indicates that, also under high-osmolarity conditions, EutE-mediated acetate reduction could not efficiently compete for NADH with a fully functional glycerol pathway.

**Table 4:** Specific growth rates (*μ*), yields (*Y*) of biomass, ethanol and glycerol on glucose and stoichiometric relationships between glycerol production and biomass formation, acetate consumption and glucose consumption, and acetate consumption and biomass formation in anaerobic bioreactor batch cultures of *S. cerevisiae* strains with different genetic modifications in glycerol and acetate metabolism

Strain	IMX992	IMX884	IMX776	IMX901
Relevant genotype	*GPD1 GPD2* *sga1::eutE*	*GPD1* *gpd2::eutE*	*gpd1::gpsA* *gpd2::eutE*	*gpd1::gpsA* *gpd2::eutE* *ald6Δ*
*μ* (h^−1^)	0.28 ± 0.02	0.27 ± 0.00	0.14 ± 0.00	0.12 ± 0.02
*Y* biomass/glucose (g g^−1^)	0.087 ± 0.001	0.085 ± 0.000	0.089 ± 0.000	0.077 ± 0.013
*Y* ethanol/glucose (g g^−1^)	0.43 ± 0.01	0.42 ± 0.02	0.47 ± 0.01	0.49 ± 0.00
*Y* glycerol/glucose (g g^−1^)	0.07 ± 0.00	0.05 ± 0.00	0.02 ± 0.00	<0.001
Glycerol produced/biomass [mmol (g biomass)^−1^]	8.76 ± 0.25	6.34 ± 0.26	3.29 ± 0.41	<0.1
Acetate consumed/biomass [mmol (g biomass)^−1^]	2.67 ± 0.96	2.98 ± 0.08	2.88 ± 0.17	5.71 ± 0.15
Acetate consumed/glucose (g g^−1^)	0.011 ± 0.001	0.015 ± 0.000	0.016 ± 0.000	0.027 ± 0.003

Cultures were grown on synthetic medium containing 180 g L^−1^ glucose and 3 g L^−1^ acetic acid (pH 5). Specific growth rates and stoichiometries were calculated from the mid-exponential growth phase and represent averages ± mean deviations of data obtained from independent duplicate cultures

Strain IMX884 (*GPD1 gpd2::eutE*) showed a 10% lower specific growth rate in high-osmolarity medium than in cultures grown on a low glucose concentration (Tables 3, 4). This, only minor, difference is consistent with the reported predominant role of the Gpd1 isoenzyme in osmoregulation [20, 21, 57]. Relative to its performance in low-osmolarity cultures, growth on 1 mol L^−1^ glucose led to a threefold increase in extracellular glycerol production [(6.34 mmol (g biomass)^−1^ versus 1.92 mmol (g biomass)^−1^] and a corresponding decrease in acetate consumption [2.98 mmol (g biomass)^−1^ versus 5.77 mmol (g biomass)^−1^] (Tables 3, 4). These changes largely eliminated the fourfold difference in glycerol production between strains IMX992 and IMX884 that was observed in low-osmolarity cultures (Tables 3, 4). After complete glucose consumption, concentrations of acetic acid, glycerol and ethanol reached similar concentrations in high-osmolarity cultures of the two strains (Fig. 3). These results indicate that, even when *GPD2* is deleted, high-osmolarity conditions impeded the competition of the EutE-based acetate-reduction pathway for NADH with the glycerol pathway, possibly due to osmotic-stress-induced upregulation of *GPD1*.

### Replacement of *GPD1* and *GPD2* by *gpsA* uncouples the roles of glycerol formation in redox metabolism and osmoregulation

To test whether replacement of the yeast NAD^+^-dependent Gpd isoenzymes by an NADP^+^-preferring G3PDH can uncouple the roles of glycerol formation in osmoregulation and redox metabolism, the growth and product formation of strain IMX776 (*gpd1::gpsA gpd2::eutE*) were investigated in high-osmolarity cultures. In contrast to strains IMX992 and IMX884, strain IMX776 showed a lag phase of ca. 50 h under these conditions (Fig. 3; Additional file 4), and its specific growth rate was 60% lower than that in low-osmolarity cultures (Tables 3, 4). While, under low-osmolarity conditions, this strain did not produce extracellular glycerol, high-osmolarity batch cultures showed a glycerol production of 3.29 mmol (g biomass)^−1^ (Table 4). After glucose depletion, the glycerol concentration in high-osmolarity cultures of strain IMX776 was 44% lower than observed for strain IMX992 (*GPD1 GPD2 sga1::eutE*) (Fig. 3).

Strain IMX776 showed a much lower acetate consumption in the high-glucose cultures than in low-osmolarity cultures (Tables 3, 4). This difference could be caused by an increased flux through the cytosolic, NADP^+^-dependent acetaldehyde dehydrogenase Ald6, coupled to the increased demand for NADPH in the cytosolic GpsA reaction. Generating NADPH via the oxidation of acetaldehyde to acetate, which can subsequently be reduced to ethanol via acetyl-CoA synthetase, EutE and NAD^+^-dependent alcohol dehydrogenase, would result in less extracellular acetate being consumed for NADH reoxidation (Additional file 8). An increased production of acetate has been previously observed upon an increase of cytosolic NADPH demand in anaerobic *S. cerevisiae* cultures [29].

Consistent with the hypothesis outlined above, deletion of *ALD6* had a strong impact on the physiology of anaerobic cultures of acetate-reducing *gpsA*-expressing *S. cerevisiae*. Although the specific growth rates of strain IMX776 (*gpd1::gpsA gpd2:eutE*) and strain IMX901 (*gpd1::gpsA gpd2:eutE ald6Δ*) in high-osmolarity cultures were similar (Table 4), complete absence of a lag phase decreased the overall fermentation time of the latter strain by ca. 35 h (Fig. 3; Additional file 4). In addition, strain IMX901 fully relied on exogenous acetic acid supply for its redox balancing. When, after exponential growth was finished, no additional acetate was provided, growth and glucose consumption slowed down considerably (Additional file 5). A similar addition of acetate to a high-osmolarity batch culture of strain IMX776 did not affect its growth (Additional file 5).

In contrast to strains IMX884 and IMX776, strain IMX901 retained a glycerol non-producing phenotype throughout growth in bioreactor cultures on high-osmolarity medium, resulting in a 13% higher ethanol yield on glucose compared to strain IMX992 (*GPD1 GPD2 sga1::eutE*; Table 4). This, in combination with a measured intracellular glycerol concentration of 5.3 ± 0.04 g L^−1^ in anaerobic shake-flask cultures of strain IMX901 on high-osmolarity medium, indicated a complete intracellular retention of glycerol formed via GpsA in this strain. When additional acetate was added to high-osmolarity bioreactor cultures of strain IMX901 immediately after the exponential phase, no extracellular glycerol was detectable (Fig. 3). However, when acetate was added 20 h into the stationary phase (Additional file 5), low concentrations of glycerol were detectable (<1 g L^−1^ final concentration).

### Growth of an acetate-reducing *gpd1Δ gpd2Δ* strain in high-osmolarity medium

In the experiments discussed above, the glycerol non-producing, acetate-reducing strain IMX888 (*gpd1Δ gpd2::eutE*) was included as a reference strain. Surprisingly, despite the absence of a functional glycerol pathway, this strain consistently grew in anaerobic high-osmolarity cultures, after a lag phase of ca. 75 h (Additional file 6). Furthermore, the strain retained its acetate-reducing phenotype, with minimal concentrations of acetate and glycerol having been produced upon glucose depletion (Additional file 7). This result contradicts earlier reports of a complete inability of *gpd1Δ gpd2Δ* strains to grow at high-osmolarity [20, 21, 43]. We therefore re-tested growth of strain IMZ160 (*gpd1Δ gpdΔ mhpF*) [43], which has been previously reported not to grow under the high-osmolarity conditions used in the present study. Consistent with previous observations, this strain failed to grow in high-osmolarity cultures, even after 300 h of incubation (Additional file 6). The different phenotypes of two acetylating-acetaldehyde expressing, glycerol-negative *S. cerevisiae* strains may reflect the lower in vivo activities of heterologously expressed MhpF relative to EutE [25, 29]. To investigate a possible involvement of *ALD6* (see above and Additional file 8), this gene was deleted in strain IMX888, yielding strain IMX900 (*gpd1Δ gpd2::eutE ald6Δ*). The latter strain showed a strongly reduced lag phase in high-osmolarity medium relative to its parental strain IMX888, thereby reducing the total fermentation time by ca. 45 h (Additional file 6). However, the overall fermentation time of strain IMX900 was still considerably longer than that of the *gpsA*-expressing *ald6Δ* strain IMX901 (Additional file 6; Fig. 3). Similar to its parental strain, IMX900 retained its acetate-reducing phenotype and produced only trace amounts of extracellular glycerol (Additional file 7). When the native *S. cerevisiae ALD6* gene, including its promoter and terminator sequences, was integrated at the *SGA1* locus of strains IMX900 and IMX901, the resulting strains (IMX1120 and IMX1142) again showed a prolonged lag phase, confirming the detrimental effect of Ald6 in this experimental context (Additional file 6; Fig. 3).

## Discussion

Expression of a heterologous acetylating-acetaldehyde dehydrogenase (A-ALD) can fully restore anaerobic growth in acetate-supplemented cultures of *S. cerevisiae* strains that lack a functional glycerol pathway [25, 29]. However, the minor decrease in glycerol formation observed upon A-ALD expression in a *GPD1 GPD2* strain (Table 3) indicated that A-ALD-based acetate reduction cannot efficiently compete for NADH with a fully functional, native glycerol pathway. Recently, a 40% decrease in glycerol yield was reported upon A-ALD expression in an industrial *S. cerevisiae* strain [58]. The higher relative impact of A-ALD expression in the industrial strain coincided with a twofold lower glycerol yield relative to that of the laboratory reference strain used in this work. These observations identify reduction of the capacity of the native glycerol pathway as an interesting strategy for facilitating NADH reoxidation via acetate reduction. Indeed, deletion of *GPD2*, which encodes the major isoenzyme of G3PDH in anaerobic, low-osmolarity cultures of *S. cerevisiae* [21, 23] strongly stimulated acetate reduction (Table 3). By enabling an over fourfold lower glycerol yield and corresponding increase in ethanol production by acetate reduction, without any reduction in specific growth rate, combined deletion of *GPD2* and expression of an A-ALD offers a promising strategy for improving ethanol production in low-osmolarity, acetic-acid containing media. In such processes, osmolarity may be limited by fed-batch cultivation regimes and/or by simultaneous saccharification and fermentation of polymeric feedstocks [59, 60]. Reducing the capacity of the glycerol pathway may be similarly effective in other metabolic engineering strategies for redirecting NADH reoxidation in anaerobic yeast cultures, such as the use of CO_2_ by Calvin-cycle-enzyme expressing yeast cultures [24], and for expression of A-ALD in engineered xylose-consuming *S. cerevisiae* strains based on expression of heterologous xylose reductase and xylitol dehydrogenase enzymes [26].

Compared to its strong impact under low-osmolarity conditions, deletion of *GPD2* had a much smaller effect on glycerol production and acetate reduction in cultures of A-ALD-expressing *S. cerevisiae* grown on 1 mol L^−1^ glucose (Table 4). This difference probably reflects the extensively documented, strong upregulation of *GPD1* under hyperosmotic stress [20, 61, 62], which is at least partly controlled by the Hog1 MAP-kinase cascade [20, 63]. Together with the increased intracellular glycerol retention [17, 64], upregulation of G3PDH activity plays a key role in the yeast osmotic stress response. The dual role of G3PDH enzymes in redox homeostasis and osmotolerance represents a challenge in redirecting NADH reoxidation in high-osmolarity, anaerobic yeast cultures towards acetate reduction. The results presented here provide a proof of principle for separating the roles of glycerol production in NADH reoxidation and osmotolerance by exchanging the native NAD^+^-dependent G3PDH enzymes for a heterologous, NADP^+^-preferring enzyme (*A. fulgidus* GpsA). In contrast to a *gpd1Δ gpd2Δ* A-ALD-expressing strain, the resulting strain was, after a lag phase, able to grow anaerobically on 1 mol L^−1^ glucose and showed an almost twofold lower glycerol yield than a *GPD1 GPD2* reference strain (Table 4). Additional deletion of *ALD6* eliminated the lag phase as well as extracellular glycerol production, yielding a strain with stoichiometric acetate consumption and ca. 13% higher ethanol yield on glucose in high-osmolarity cultures than observed for a *GPD1 GPD2* reference strain (Table 4). This ethanol yield improvement was consistent with the results obtained in studies on glucose-grown, acetate-reducing strains that were not further engineered for osmotolerance [25, 29, 30] and in an osmotolerant acetate-reducing strain obtained after prolonged laboratory evolution [43].

Several factors may explain the strong impact of deleting *ALD6* on high-osmolarity cultures. In glucose-grown cultures of wild-type *S. cerevisiae* strains, NADP^+^-dependent oxidation of acetaldehyde to acetate by Ald6 can account for ca. 20% of the total cytosolic NADPH requirement [65, 66]. Even possibly higher contributions of Ald6 have been reported in genetic backgrounds that affect NADPH supply via other pathways [29, 67]. In *gpsA*-expressing strains, NADPH generated by acetate formation via Ald6 can directly contribute to glycerol production. Since, during growth on glucose, NADPH formation by Ald6 is coupled to equimolar generation of NADH in glycolysis, the increased acetate formation via Ald6 can help meet an increased NADH demand for glycerol production via Gpd1 and/or Gpd2 during hyper-osmotic stress [20, 63]. Furthermore, Ald6, together with yeast acetyl-CoA synthetases (Acs1, Acs2) and heterologously expressed A-ALD, have been proposed to form an ATP-driven transhydrogenase cycle [29]. Activity of such a cycle in high-osmolarity cultures, possibly stimulated by upregulation of *ALD6*, could impose an ATP drain that impedes growth under the combined stresses of high osmolarity and acetate uncoupling. Elimination of such an ATP drain could explain why the deletion of *ALD6* eliminated lag phases in high-osmolarity, acetate-supplemented cultures. Increased adaptation phases, reflecting a non-genetic population heterogeneity, are well documented in acetate-stressed *S. cerevisiae* cultures [68, 69]. Recent studies on engineered A-ALD expressing *S. cerevisiae* strains in low- and medium-osmolarity cultures [26, 29] lend further support to the conclusion that deletion of *ALD6* is a key step in engineering efficient pathways for acetate reduction in *S. cerevisiae*.

While a *gpd1Δ gpd2Δ ald6Δ* strain expressing *gpsA* and *eutE* showed an excellent stoichiometry in terms of ethanol yield and acetate reduction, its growth rate in high-osmolarity cultures was substantially lower than that of strains expressing *GPD1* and/or *GPD2*. To minimize costs of yeast propagation and to maximize ethanol productivity, a high maximum specific growth rate is important for application in lignocellulosic ethanol production. As previously demonstrated for a *gpd1Δ gpd2Δ* strain expressing *mhpF* [43], evolutionary engineering can enable selection for faster-growing mutants. Alternatively, growth kinetics may be improved by optimizing the expression levels of NADPH-dependent G3PDH and/or by improving the availability of cytosolic NADPH [65, 66].

In view of the extensively documented, central role of G3PDH in the hyperosmotic-stress response of *S. cerevisiae* [17, 70], the slow but reproducible anaerobic growth of a *gpd1Δ gpd2Δ ald6Δ*, *eutE*-expressing *S. cerevisiae* strain in a medium containing 1 mol L^−1^ glucose was an unexpected result. In addition to possible contributions of the *ald6Δ* mutation and/or *eutE* expression to osmotolerance, the lag phases of *gpd1Δ gpd2Δ* strains in high-osmolarity cultures may have obscured this interesting phenotype in previous short-term growth studies [20, 21]. Osmotolerance in *S. cerevisiae* is a complex, multi-gene phenotype [71] and, especially upon sudden exposure to osmotic stress, G3PDH-independent mechanisms have been proposed to contribute to osmotolerance [64, 72], such as trehalose accumulation in osmotically challenged cultures growing on galactose [73]. Alternatively, intracellular glycerol could be derived via a G3PDH-independent pathway by de-acylation of acyl-glycerol-3-phosphate, which can be formed from dihydroxyacetone phosphate (DHAP) by the combined activities of DHAP acyltransferase and NADPH-linked 1-acylglycerol-3-phosphate acyltransferase [74]. Activity of the latter pathway may explain low levels of glycerol production in an A-ALD-expressing *gpd1Δ gpd2Δ* strain evolved for increased osmotolerance [43]. The acetate-reducing, *gpd1Δ gpd2Δ* strains constructed in this work provide an interesting experimental platform for further fundamental research on G3PDH-independent mechanisms for osmotolerance in *S. cerevisiae*.

## Conclusions

Deletion of *GPD2* provides a straightforward engineering strategy for maximizing the positive impact of A-ALD-based, engineered pathways in low-osmolarity cultures of *S. cerevisiae*, by improving acetate conversion and ethanol yields. Replacement of the NAD^+^-dependent *S. cerevisiae* glycerol-3P dehydrogenases by a heterologous NADP^+^-dependent enzyme enables uncoupling of the function of glycerol as an osmoprotectant from its role in cellular redox-cofactor balancing. When combined with a deletion of *ALD6*, thereby eliminating the influence of cytosolic NADP^+^-dependent acetaldehyde oxidation on redox metabolism, this engineering strategy enables anaerobic growth and efficient acetate reduction, with its associated improvement of ethanol yields, in high-osmolarity cultures. If the growth kinetics of the resulting strains can be further improved, this approach is highly promising for application in high-gravity processes for conversion of acetate-containing, lignocellulosic hydrolysates.

## Additional files

**Additional file 1.** Primers used in this study.

**Additional file 2.** Specific rates of EutE-dependent reduction of acetyl-CoA by cell extracts of shake-flask cultures on synthetic medium (20 g L^−1^) glucose. From left to right: *S. cerevisiae* strains IMX992 (*GPD1 GPD2 sga1::eutE*), IMX884 (*GPD1 gpd2::eutE*) and IMX776 (*gpd1::gpsA gpd2::eutE*). Data represent averages ± mean deviations of assays on independent duplicate cultures.

**Additional file 3.** Biomass and product yields in anaerobic bioreactor batch cultures of *S. cerevisiae* strains with different genetic modifications in glycerol and acetate metabolism. Cultures were grown on synthetic medium containing 20 g L^−1^ glucose and 3 g L^−1^ acetic acid (pH 5). Bars refer to the following engineered *S. cerevisiae* strains: IME324 (*GPD1 GPD2*); IMX992 (*GPD1 GPD2 sga1::eutE*); IMX884 (*GPD1 gpd2::eutE*); IMX776 (*gpd1::gpsA gpd2::eutE*); IMX901 (*gpd1::gpsA gpd2::eutE ald6Δ*); IMX888 (*gpd1Δ gpd2::eutE*). A, biomass yield on glucose; B, ethanol yield on glucose (corrected for ethanol evaporation); C, glycerol yield on glucose. Data represent the averages ± mean deviations of measurements on independent duplicate cultures for each strain. Data on strain IMX888 were taken from [29].

**Additional file 4.** Plots of ln(OD_660_) values versus time in anaerobic bioreactor batch cultures of *S. cerevisiae* strains with different genetic modifications in glycerol and acetate metabolism (from inoculation to mid-exponential phase). Cultures were grown on synthetic medium containing 180 g L^−1^ glucose and 3 g L^−1^ acetic acid (pH 5). ▪, strain IMX992 (*GPD1 GPD2 sga1::eutE*); ▫, strain IMX884 (*GPD1 gpd2::eutE*); ◊, strain IMX776 (*gpd1::gpsA gpd2::eutE*); ∆, strain IMX901 (*gpd1::gpsA gpd2::eutE ald6Δ*). The figure shows representative cultures of independent duplicate experiments.

**Additional file 5.** Growth, glucose consumption and product formation in anaerobic bioreactor batch cultures of *S. cerevisiae* strains with different genetic modifications in glycerol and acetate metabolism. Cultures were grown on synthetic medium containing 180 g L^−1^ glucose and 3 g L^−1^ acetic acid (pH 5). A, strain IMX776 (*gpd1::gpsA gpd2::eutE*); B, strain IMX901 (*gpd1::gpsA gpd2::eutE ald6Δ*). Symbols: ●, glucose; ▪, biomass; □, glycerol; ○, ethanol; Δ, acetate. In the case of IMX776, acetic acid was added externally immediately after the exponential growth phase was finished. In the case of IMX901, acetic acid was added externally after 20 h in stationary phase.

**Additional file 6.** CO_2_ production profiles in anaerobic bioreactor batch cultures of *S. cerevisiae* strains with different genetic modifications in glycerol and acetate metabolism. Cultures were grown on synthetic medium containing 180 g L^−1^ glucose and 3 g L^−1^ acetic acid (pH 5). A, IMZ160 (*gpd1::loxP gpd2::hphMX4 mhpF*–overexpressing); B, IMX888 (*gpd1Δ gpd2::eutE*); C, IMX900 (*gpd1Δ gpd2::eutE ald6Δ*); D, IMX1120 (*gpd1Δ gpd2::eutE ald6Δ sga1::ALD6*); E, IMX1142 (*gpd1::gpsA gpd2::eutE ald6Δ sga1::ALD6*). Data collected from online bioreactor offgas measurements. Representative cultures of independent duplicate experiments are shown.

**Additional file 7.** Starting and end concentrations of acetate and glycerol in anaerobic bioreactor batch cultures of *S. cerevisiae* strains IMX888 (*gpd1Δ gpd2::eutE*) and IMX900 (*gpd1Δ gpd2::eutE ald6Δ*). Cultures were grown on synthetic medium containing 180 g L^−1^ glucose and 3 g L^−1^ acetate (pH 5). Values represent averages ± mean deviations of measurements on independent duplicate cultures.

**Additional file 8.** Potential cytosolic transhydrogenase cycle, exchanging NADH with NADPH, catalysed by EutE, Acs1/2 and Ald6. Formed NADPH can be used for DHAP reduction to glycerol by GpsA.

## Abbreviations

G3PDH: glycerol-3-phosphate dehydrogenase
KPB: potassium-phosphate buffer
OD: optical density
A-ALD: acetylating-acetaldehyde dehydrogenase
