# Supplementary material for: Metabolic engineering strategies for optimizing acetate reduction, ethanol yield and osmotolerance in Saccharomyces cerevisiae
Source: Biotechnol Biofuels. 2017 Apr 26;10:107. doi: 10.1186/s13068-017-0791-3 (PMC5406903; doi:10.1186/s13068-017-0791-3)
Supplement: Supplementary file 2 — Additional file 2. Specific rates of EutE-dependent reduction of acetyl-CoA by cell extracts of shake-flask cultures on synthetic medium (20 g L−1) glucose. From left to right: S. cerevisiae strains IMX992 (GPD1 GPD2 sga1::eutE), IMX884 (GPD1 gpd2::eutE) and IMX776 (gpd1::gpsA gpd2::eutE). Data represent averages ± mean deviations of assays on independent duplicate cultures. [file 13068_2017_791_MOESM2_ESM.docx]

Additional File S2.
