# Supplementary material for: Metabolic engineering strategies for optimizing acetate reduction, ethanol yield and osmotolerance in Saccharomyces cerevisiae
Source: Biotechnol Biofuels. 2017 Apr 26;10:107. doi: 10.1186/s13068-017-0791-3 (PMC5406903; doi:10.1186/s13068-017-0791-3)
Supplement: Supplementary file 3 — Additional file 3. Biomass and product yields in anaerobic bioreactor batch cultures of S. cerevisiae strains with different genetic modifications in glycerol and acetate metabolism. Cultures were grown on synthetic medium containing 20 g L−1 glucose and 3 g L−1 acetic acid (pH 5). Bars refer to the following engineered S. cerevisiae strains: IME324 (GPD1 GPD2); IMX992 (GPD1 GPD2 sga1::eutE); IMX884 (GPD1 gpd2::eutE); IMX776 (gpd1::gpsA gpd2::eutE); IMX901 (gpd1::gpsA gpd2::eutE ald6Δ); IMX888 (gpd1Δ gpd2::eutE). A, biomass yield on glucose; B, ethanol yield on glucose (corrected for ethanol evaporation); C, glycerol yield on glucose. Data represent the averages ± mean deviations of measurements on independent duplicate cultures for each strain. Data on strain IMX888 were taken from [29]. [file 13068_2017_791_MOESM3_ESM.docx]

Additional File S3.
