# Supplementary material for: Metabolic engineering strategies for optimizing acetate reduction, ethanol yield and osmotolerance in Saccharomyces cerevisiae
Source: Biotechnol Biofuels. 2017 Apr 26;10:107. doi: 10.1186/s13068-017-0791-3 (PMC5406903; doi:10.1186/s13068-017-0791-3)
Supplement: Supplementary file 4 — Additional file 4. Plots of ln(OD660) values versus time in anaerobic bioreactor batch cultures of S. cerevisiae strains with different genetic modifications in glycerol and acetate metabolism (from inoculation to mid-exponential phase). Cultures were grown on synthetic medium containing 180 g L−1 glucose and 3 g L−1 acetic acid (pH 5). ▪, strain IMX992 (GPD1 GPD2 sga1::eutE); ▫, strain IMX884 (GPD1 gpd2::eutE); ◊, strain IMX776 (gpd1::gpsA gpd2::eutE); ∆, strain IMX901 (gpd1::gpsA gpd2::eutE ald6Δ). The figure shows representative cultures of independent duplicate experiments. [file 13068_2017_791_MOESM4_ESM.docx]

Additional File S4.
