# Supplementary material for: Metabolic engineering strategies for optimizing acetate reduction, ethanol yield and osmotolerance in Saccharomyces cerevisiae
Source: Biotechnol Biofuels. 2017 Apr 26;10:107. doi: 10.1186/s13068-017-0791-3 (PMC5406903; doi:10.1186/s13068-017-0791-3)
Supplement: Supplementary file 5 — Additional file 5. Growth, glucose consumption and product formation in anaerobic bioreactor batch cultures of S. cerevisiae strains with different genetic modifications in glycerol and acetate metabolism. Cultures were grown on synthetic medium containing 180 g L−1 glucose and 3 g L−1 acetic acid (pH 5). A, strain IMX776 (gpd1::gpsA gpd2::eutE); B, strain IMX901 (gpd1::gpsA gpd2::eutE ald6Δ). Symbols: ●, glucose; ▪, biomass; □, glycerol; ○, ethanol; Δ, acetate. In the case of IMX776, acetic acid was added externally immediately after the exponential growth phase was finished. In the case of IMX901, acetic acid was added externally after 20 h in stationary phase. [file 13068_2017_791_MOESM5_ESM.docx]

Additional File S5.
