# Supplementary material for: Metabolic engineering strategies for optimizing acetate reduction, ethanol yield and osmotolerance in Saccharomyces cerevisiae
Source: Biotechnol Biofuels. 2017 Apr 26;10:107. doi: 10.1186/s13068-017-0791-3 (PMC5406903; doi:10.1186/s13068-017-0791-3)
Supplement: Supplementary file 6 — Additional file 6. CO2 production profiles in anaerobic bioreactor batch cultures of S. cerevisiae strains with different genetic modifications in glycerol and acetate metabolism. Cultures were grown on synthetic medium containing 180 g L−1 glucose and 3 g L−1 acetic acid (pH 5). A, IMZ160 (gpd1::loxP gpd2::hphMX4 mhpF–overexpressing); B, IMX888 (gpd1Δ gpd2::eutE); C, IMX900 (gpd1Δ gpd2::eutE ald6Δ); D, IMX1120 (gpd1Δ gpd2::eutE ald6Δ sga1::ALD6); E, IMX1142 (gpd1::gpsA gpd2::eutE ald6Δ sga1::ALD6). Data collected from online bioreactor offgas measurements. Representative cultures of independent duplicate experiments are shown. [file 13068_2017_791_MOESM6_ESM.docx]

Additional File S6.
