# Supplementary material for: Metabolic engineering strategies for optimizing acetate reduction, ethanol yield and osmotolerance in Saccharomyces cerevisiae
Source: Biotechnol Biofuels. 2017 Apr 26;10:107. doi: 10.1186/s13068-017-0791-3 (PMC5406903; doi:10.1186/s13068-017-0791-3)
Supplement: Supplementary file 7 — Additional file 7. Starting and end concentrations of acetate and glycerol in anaerobic bioreactor batch cultures of S. cerevisiae strains IMX888 (gpd1Δ gpd2::eutE) and IMX900 (gpd1Δ gpd2::eutE ald6Δ). Cultures were grown on synthetic medium containing 180 g L−1 glucose and 3 g L−1 acetate (pH 5). Values represent averages ± mean deviations of measurements on independent duplicate cultures. [file 13068_2017_791_MOESM7_ESM.docx]

Additional File S7.

| **Strain** | **Acetate g L^-1^** | | **Glycerol g L^-1^** | |
| --- | --- | --- | --- | --- |
|  | **Start** | **End** | **Start** | **End** |
| **IMX888** | 3.26 ± 0.02 | 0 | 0.12 ± 0.00 | 0.30 ± 0.00 |
| **IMX900** | 2.79 ± 0.08 | 0 | 0.11 ± 0.00 | 0.42 ± 0.00 |
