# Supplementary figures and images for: Metabolic engineering strategies for optimizing acetate reduction, ethanol yield and osmotolerance in Saccharomyces cerevisiae
Source: Biotechnol Biofuels. 2017 Apr 26;10:107. doi: 10.1186/s13068-017-0791-3 (PMC5406903; doi:10.1186/s13068-017-0791-3)

Additional File S8.


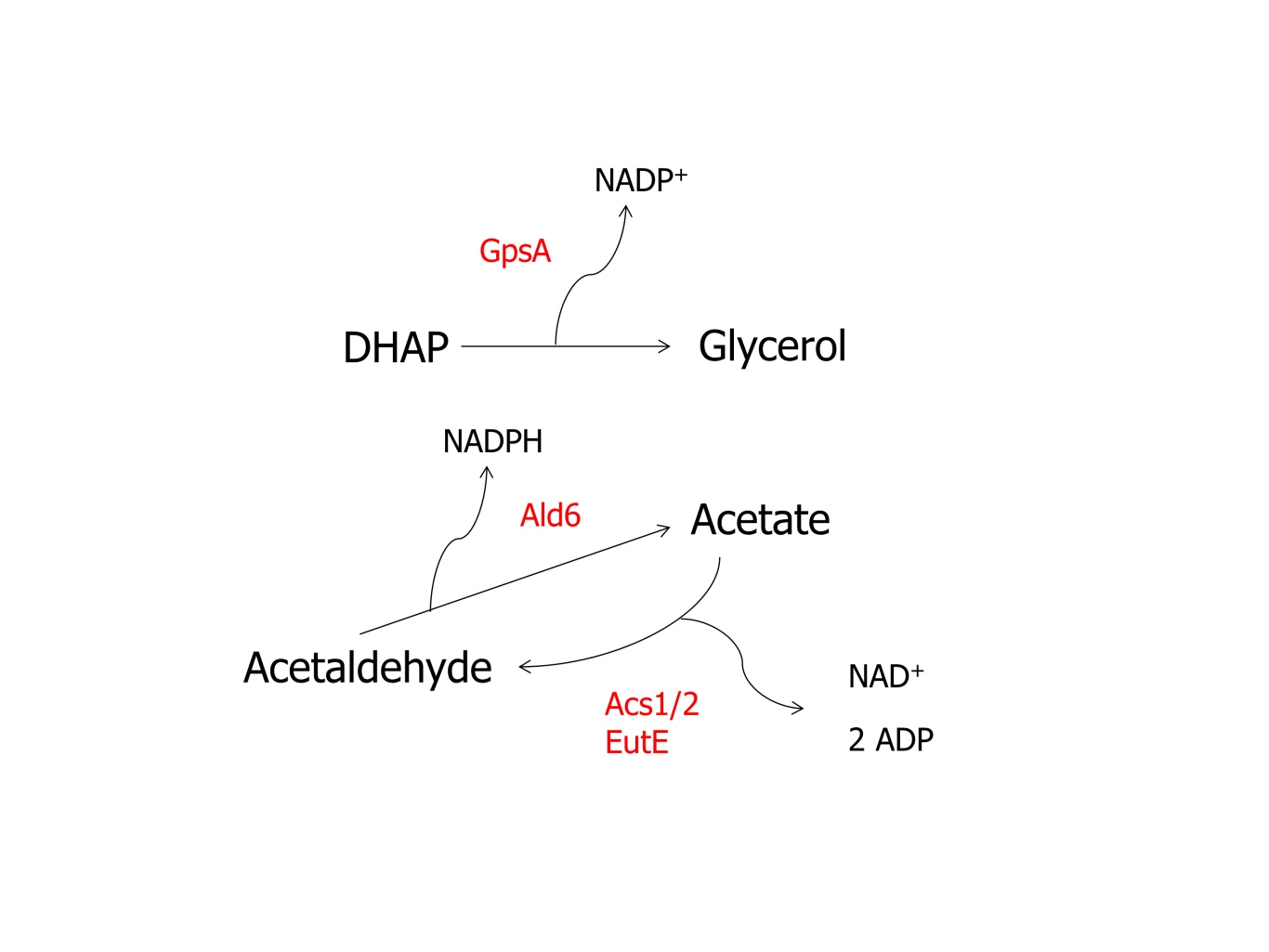

Supplement: Supplementary file 8 — Additional file 8. Potential cytosolic transhydrogenase cycle, exchanging NADH with NADPH, catalysed by EutE, Acs1/2 and Ald6. Formed NADPH can be used for DHAP reduction to glycerol by GpsA. [file 13068_2017_791_MOESM8_ESM.docx]
